# A user needs assessment to inform health information exchange design and implementation

**DOI:** 10.1186/s12911-015-0207-x

**Published:** 2015-10-12

**Authors:** Alexandra T. Strauss, Diego A. Martinez, Andres Garcia-Arce, Stephanie Taylor, Candice Mateja, Peter J. Fabri, Jose L. Zayas-Castro

**Affiliations:** Department of Internal Medicine, College of Medicine, University of South Florida, Tampa, FL USA; Johns Hopkins Department of Emergency Medicine, Baltimore, MD USA; Department of Industrial and Management Systems Engineering, College of Engineering, University of South Florida, Tampa, FL USA; Department of Internal Medicine, Carolinas Medical Center, Charlotte, NC USA; Department of Surgery, College of Medicine, University of South Florida, Tampa, FL USA

**Keywords:** Health information technology, Health information exchange, Medical decision making, Hospital medicine, Medical record linkage, Computer communication networks, Continuity of patient care, Care coordination

## Abstract

**Background:**

Important barriers for widespread use of health information exchange (HIE) are usability and interface issues. However, most HIEs are implemented without performing a needs assessment with the end users, healthcare providers. We performed a user needs assessment for the process of obtaining clinical information from other health care organizations about a hospitalized patient and identified the types of information most valued for medical decision-making.

**Methods:**

Quantitative and qualitative analysis were used to evaluate the process to obtain and use outside clinical information (OI) using semi-structured interviews (16 internists), direct observation (750 h), and operational data from the electronic medical records (30,461 hospitalizations) of an internal medicine department in a public, teaching hospital in Tampa, Florida.

**Results:**

13.7 % of hospitalizations generate at least one request for OI. On average, the process comprised 13 steps, 6 decisions points, and 4 different participants. Physicians estimate that the average time to receive OI is 18 h. Physicians perceived that OI received is not useful 33–66 % of the time because information received is irrelevant or not timely. Technical barriers to OI use included poor accessibility and ineffective information visualization. Common problems with the process were receiving extraneous notes and the need to re-request the information. Drivers for OI use were to trend lab or imaging abnormalities, understand medical history of critically ill or hospital-to-hospital transferred patients, and assess previous echocardiograms and bacterial cultures. About 85 % of the physicians believe HIE would have a positive effect on improving healthcare delivery.

**Conclusions:**

Although hospitalists are challenged by a complex process to obtain OI, they recognize the value of specific information for enhancing medical decision-making. HIE systems are likely to have increased utilization and effectiveness if specific patient-level clinical information is delivered at the right time to the right users.

**Electronic supplementary material:**

The online version of this article (doi:10.1186/s12911-015-0207-x) contains supplementary material, which is available to authorized users.

## Background

In the United States, 125 million people live with chronic conditions [1], and most of them receive care from multiple health care providers [2]. For these patients, care coordination is a necessity. Without care coordination, patients may undergo avoidable procedures, receive contraindicated treatments and incur unnecessary costs [3, 4]. To foster care coordination, federal incentives have been in place since 2009 to promote health information exchange (HIE). HIE refers to the electronic movement of health-related information among health care organizations intended to facilitate a safer and more timely, efficient, effective and equitable delivery of care [5].

Mixed evidence supports the ability of HIE to add value to healthcare systems [6, 7], to detect patient safety issues [8, 9] and to reduce healthcare delivery time and redundant testing [10–16]. For instance, Bailey and colleagues found HIE reduces repeated imaging testing for back pain and headache admissions in emergency departments, but has a negligible effect on reducing costs [11, 12]. Frisse and colleagues found a negative association between HIE usage and hospital admissions, computerized tomography (CT) scans and laboratory tests [17]. Vest and Miller found better patient satisfaction levels in those hospitals with HIE versus those without HIE [18]. Nguyen and colleagues reported a perceived need by healthcare providers and social service providers for improved health information sharing [19]. In contrast, Overhage and colleagues found no significant effect of HIE on reducing testing and number of admissions [13]. Lang and colleagues found HIE use associated with duplication of specialty consultations, as well as no significant effect of HIE on reducing number of hospital admissions, length of stay and number of tests [20]. Finally, Hansagi and colleagues found HIE use improved physician satisfaction, but no significant effects were observed on the number of emergency department, primary care and specialty visits [21]. A potential reason for the mixed evidence, as suggested by recently published systematic reviews [6, 7], is that widespread adoption of HIE across the United States is still limited. To date, only 14 % of solo practices and non-primary care specialties, 30 % of hospitals, and 10 % of ambulatory clinics are engaged in an HIE, with typical rates of access from 2 to 10 % of patient visits [22–24]. Despite substantial progress in electronic medical record (EMR) adoption, physician engagement in HIE remains low in office settings [24].

Research revealing how health professionals use HIE systems to obtain information from other institutions can help improve HIE functionality and subsequently improve HIE utilization. Some have explored the user’s interaction in ambulatory care situations [25]. Although early studies concentrated on identifying drivers and barriers for HIE adoption [18, 25–28], recent studies have shed light on HIE use patterns. For example, it has been found that physicians are more likely to access radiology reports than any other health professional [29, 30], and that all users engage with HIE systems in a minimal fashion by accessing only the select patient screen and the recent encounters summary screen [31]. Additionally, it has been shown that time constraints are an important barrier to HIE usage [27, 28, 32–34], which might result in health professionals being reluctant to engage in HIE. Based on these results, we suggest that tailoring the type of information displayed on the first screens of HIE systems by type of user (e.g., physician, nurse) and discipline (e.g., emergency medicine, pediatrics) might improve HIE utilization by providers. Furthermore, most prior studies were performed in emergency departments with providers already using HIE. New products often benefit from a user needs assessment before, during, and after the development cycle. We believe HIE systems will be more successful if they are developed with *a priori* input from its future users. Our work is unique as it provides a clinician needs assessment prior to HIE implementation, so the providers have not developed biases of using an HIE. Furthermore, our research expands the current evidence by focusing on an unexplored clinical setting in regards to HIE: an Internal Medicine (IM) Hospitalist Department.

In this study, we investigated an IM Department in a teaching hospital in Tampa, Florida before HIE implementation. Our objectives were to understand the process of obtaining medical information from other facilities prior to HIE, explore provider perceptions of the usage of outside information for medical decision-making, and to analyze their views on the potential impact of HIE. Improving HIE developers’, policy makers’, and administrators’ understandings about how documents from outside institutions, referred to as outside information (OI), are collected and utilized by clinicians can inform HIE design and implementation which could improve HIE usability.

## Methods

We used a convergent mixed-methods study design to gather insights about the performance of the current fax-based process to request OI, the use of OI for medical-decision making, and the physicians’ perceptions of HIE implementation. We conducted semi-structured interviews with both IM third-year residents and attending physicians and performed direct observation of the workflows in the IM Department. In addition, we collected demographic and clinical data of hospitalizations that generated at least one request for OI. Institutional review board approval was granted for this study by the hospital’s Office of Clinical Research and the University of South Florida (IRB Number: Pro00014574).

### Study setting and datasets

This research was performed in the IM Department of a public, teaching hospital in Tampa, Florida. The hospital is a 1018-bed hospital serving 23 counties in Tampa using the electronic medical record system (EMR) Epic (EpiCare; Verona, WI) with no HIE functionality enabled. We considered three sources of data: direct observation, interviews, and the EMR. First, we observed approximately 750 h of the workflows and medical decision-process related to the request of OI. Second, we interviewed resident and attending physicians from the IM Department from January to February 2014. Finally, from the hospital’s EMR, we extracted demographic and clinical factors for each hospitalization from October 2011 to March 2014 that generated at least one request for OI. We also extracted operational data related to the request for OI: timestamps for the request and receipt of OI, type of health professional requesting OI, and type of information received.

### Process mapping

We followed a two-step method of observation and validation to document the process to request and collect OI. We created a process chart that represents the activities performed, resources used, and people involved in order to obtain OI. To construct these diagrams, our team of industrial engineers and physicians observed the process and created preliminary flow process charts. During observation, the team shadowed and interviewed medical teams, nurses and personnel from the medical records department. Three people each performed 30 observation periods. During each period, between 6 and 10 h were observed. Observations were performed every day of the week and during working hours. During these observations, between 3 and 5 providers were observed on both attending and resident physicians. Observers recorded their observations when necessary. The initial flow process charts were then validated by subject matter experts, which included physicians and the medical records department. We validated the process map during semi-structured interviews with the third year residents and attending physicians until saturation. During this validation process, we discussed perceived process times and any additional comments about each step in the process.

### Interviews

A semi-structured interview (see Additional file 1) including 8 questions was performed with 16 physicians from the IM Department. All attending physicians in the IM Department and all third year resident physicians were e-mailed to be invited to participate in the study. We used a non-probabilistic convenience sampling approach. In an effort to reduce interviewer bias, a team member with expertise in interviewing methods prepared a 1-day training for the other members of the team. Additionally, the questions included in the interviews were discussed with subject experts to avoid potential bias imposed by the team. The duration of the interview was 30 min. An informed consent was reviewed and signed by each physician. Each interview was audio recorded and transcribed for posterior analysis. Afterwards, the de-identified transcripts were analyzed to code the main themes reported by the subjects using Atlas.ti version 6.0 [35]. The coding process was performed concurrently by three study members with experience in medicine, systems engineering, and qualitative analysis. In case of disagreement, the study members discussed the alternatives and a majority vote determined the final result.

## Results

### Interview respondents

Sixteen out of thirty-eight physicians participated (42.1 % response rate). The 16 study subjects included 11 third-year resident physicians and 5 attending physicians. There were an equal number of male and female subjects. On average, interviewees had been using the same EMR system for 2.5 years prior to the study. The 30-min interviews were transcribed and generated a free text document containing 37,579 words that was analyzed using Atlas.ti.

### EMR data

Table 1 describes the hospitalizations for which OI was requested. The study population was 50.7 % female and 98.2 % English speaking followed by 4.5 % Spanish speaking preference. The mean age was 53.5 years old.

**Table 1:** Demographic and clinical factors of hospitalizations with at least one request for outside information

	No. (%) *N* = 2091
Female	1061 (50.7)
Language preference	
English	1949 (93.2)
Spanish	95 (4.5)
Unknown/Other	47 (2.3)
Marital status	
Single	1361 (65.1)
Married	652 (31.2)
Unknown/Other	78 (3.7)
Primary care provider	1235 (59.1)
Payer class	
Commercial	627 (30)
Medicare	817 (39.1)
Medicaid	465 (22.2)
HCHCP	137 (6.6)
Other	45 (2.1)
Admission source	
Emergency room	1921 (91.9)
Physician-referral	84 (4)
Outside hospital	84 (4)
Other	2 (0.1)
	Mean (SD)
Age	53.5 (17.3)
Length of stay	6.7 (10)

*HCHCP* Hillsborough Country Health Care Plan

### Pre-HIE process map of obtaining OI

**Fig. 1:**
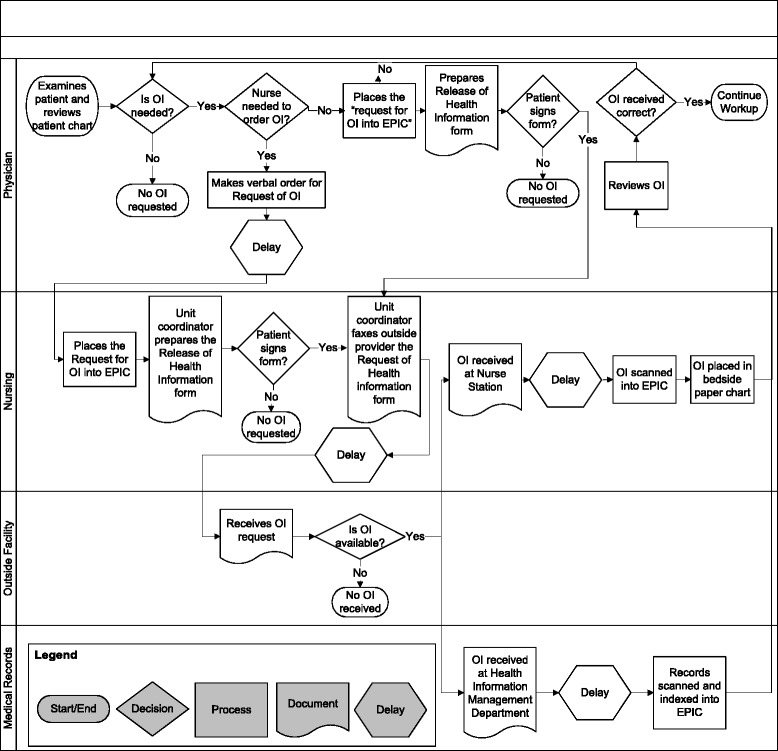
Flow process chart of obtaining outside information. Abbreviations: OI, outside information

Using the information collected from shadowing medical teams, interviewing physicians and meeting with medical records personnel, a final flow process chart was created (see Fig. 1). The boxes with curved bottoms represent steps in the process involving paper. Each step was separated depending on the person or location in which it took place. The current process to obtain outside records comprises eight steps, five paper generation steps, six decision points and at least four different personnel. The pre-HIE process flow chart demonstrates where HIE can improve the sharing of information. The process map shows that various individuals with different levels of medical expertise and in different locations are required to complete myriad steps at different times. Many steps involve paper documents to be generated and moved. For example, documents housed in one hospital need to be faxed page by page by an individual which generate another set of documents at the receiving hospital. Then, the duplicated paper documents are scanned into a computer, stored and later shredded. These actions require human and physical resources, as well as time. These types of waste could be largely replaced by a few clicks in an effectively designed HIE system.

**Fig. 2:**
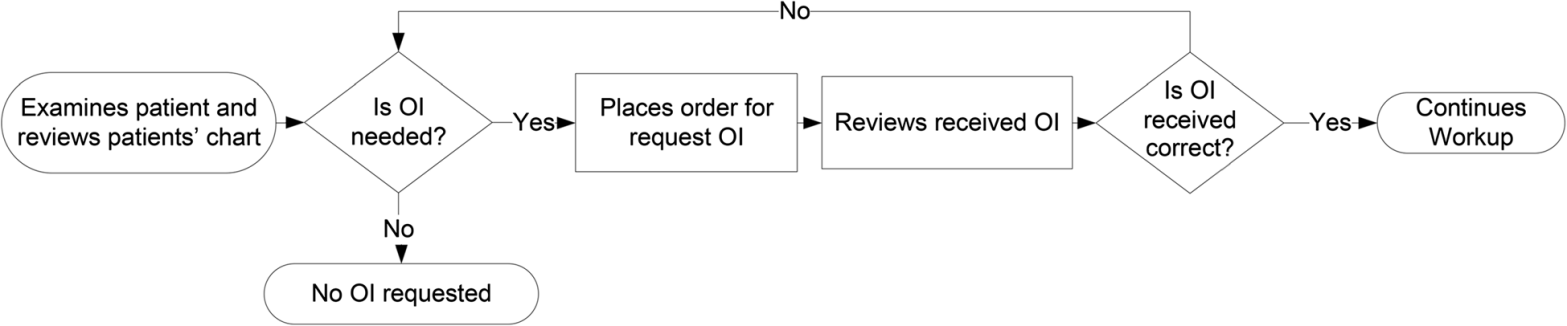
Simplified flow process chart of obtaining outside information from physician perspective

Figure 2 represents a simplified flow process chart. Physicians believed that the time between identifying the need for OI and placing the request ranges between 1 min and 5 days, with a mode of 45 min. Our evaluation on the time actual orders to obtain HIE were entered into the EMR indicated that the median delay between admission and electronic order of OI request was 10-h. This demonstrates potential time that could be saved by effective HIE implementation if information was available immediately on admission to the hospital. Physicians estimated that the time between the request and when the information was viewed ranged between 1 and 72 h, with a mode of 18 h.

The interviews revealed that providers want alerts upon the arrival of OI. We found OI is sometimes faxed directly to the nurse’s station or the hospital’s Health Information Management Department depending on what information is sent with the request. When OI arrives, physicians must wait for the OI to be scanned into the hospital EMR to have access to the information, and must repeatedly check to see if the information is available. This suggests that effective HIE designs should include a feature to alert providers once OI is available for viewing. Another insight elicited through the interviews was that physician satisfaction with the OI received was higher among those who made follow-up phone calls to outside facilities to inquire about the record request. Also, physicians specifying exactly which data items they need in the OI request improved the value of the OI received.

### Perceptions on use of OI compared to EMR data

To explore physicians’ perceptions we asked, “What percentage of your patients do you request for OI?” Most physicians believe they request outside records for 5 to 10 % of their patients. We were able to compare the provider perceptions to the quantitative data and found that out of 15,230 admissions to the IM Department during the study timeframe, 2091 generated at least one request for OI (13.7 %). In addition, we were able to explore what factors influenced when the physician did not need OI. Responses to the question, “In which situations do you know OI exists but you do not request for records?” are presented in Table 2. Most physicians answered that if the current admission is unrelated to OI (i.e., “…it may be unrelated to the acute [issue] they are coming in for.”), then they do not need that data. About 25 % of physicians reported that the process would take too long, so they did not feel it was useful to request the information (i.e., “I rarely request them because it’s so difficult to get them. But I find it is usually not worth the time.”). Most of the physicians (75 %) estimated that the information was not received or incorrect more than 33 % of the time. Our analysis of EMR data showed that in 814 out of 2091 (38.9 %) admissions, OI was requested but no documents were received.

**Table 2:** Summary of physician perceptions of current, pre-HIE use of outside information requested from outside hospitals

Reasons for not requesting	Problems encountered
1. Time	1. Process
● Outside information is too old	● Need to re-request
● Physician assumes the OI request process takes too long	● Delay in sending or scanning outside information after work hours
● Emergent situations	● Transitions-of-care communication problems
● Brief Hospital stay	● Problems with outside information transfer patients
	● Do not receive any outside information
	● OI comes too late
	● Delay waiting for imaging to be loaded from CD
	● Unaware of where outside information is in the process or if it has arrived
2. Relevance	2. Information
● Current admission unrelated to outside information	● Unhelpful physician or nursing notes
● Unnecessary to request outside information based on clinical expertise	● Difficulty finding useful information in unorganized and abundant amount of outside information
	● Skepticism of imaging or culture reads from outside facility
3. Patient	
● Patient or family is good historian and record keeper	
● Patient does not know where to request outside information from	

*OI* outside information

The majority of physicians stated that the information received is often a large amount of data that is not organized for quick clinical use. The majority of physicians believed that between 33 and 66 % of all OI received is not useful. They elaborated that they might only be looking for specific data items, but an abundance of daily monitoring notes make it difficult to find relevant information. They also reported OI was not useful because it was not the information they had requested. See Table 2 for physician responses to the prompt: “Give examples in which outside information was requested and you encountered problems. What percentage?”. This perception was compared to our findings from the data from the EMR. OI received from outside facilities are indexed as “medical record”, “imaging”, “history and physical”, “note”, “discharge summary”, “electrocardiogram”, or “consultation”. As shown in Table 3, most of the documents received were medical records (*n* = 2343) followed by imaging (*n* = 567) and history and physical (*n* = 395). Therefore, most received documents are labeled ambiguously as “medical records”, consistent with physician perceptions that the OI is usually not useful. Mitigating an overabundance of data with efficient categorization of records is key for the successful future of HIE.

**Table 3:** Document types received from outside health care facilities

Document type	Number of documents received (%) *N* = 2091
Medical record	1637 (78)
Imaging	383 (18)
History and physical	255 (12)
Note	206 (10)
Discharge summary	164 (8)
Electrocardiogram	153 (7)
Consultation	151 (7)

### Physician-identified clinical drivers for future HIE use

**Fig. 3:**
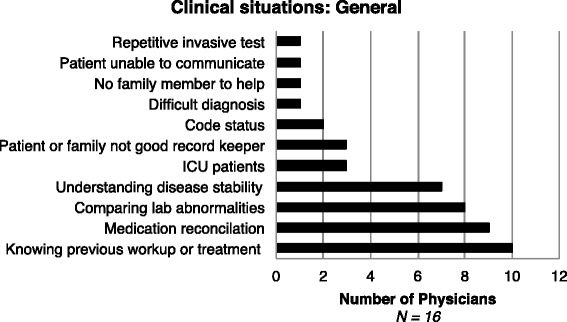
Response distribution to the question “In which specific (general) clinical situations would timely OI influence your medical decisions?” Abbreviations: ICU, intensive care unit

Through our user needs assessment, we were able to identify common themes of clinical drivers for physicians requesting OI and medical decision-making using OI. By focusing on the drivers of OI requests, HIE designers and administration can work with clinicians to give physicians information they need at a time that it is clinically relevant. Physicians were asked, “In which specific clinical situations would timely OI influence your medical decisions?”. The research team classified the clinical drivers for OI described by physicians into three groups: general, test-related, and health condition. As shown in Fig. 3, 10 out of 16 interviewed physicians reported “knowing previous workup or treatment”, “medication reconciliation” and “comparing lab abnormalities” as clinical drivers where having OI may influence medical decisions. In general, physicians found OI most beneficial if the patient was unable to communicate and information was not available from family members.

**Fig. 4:**
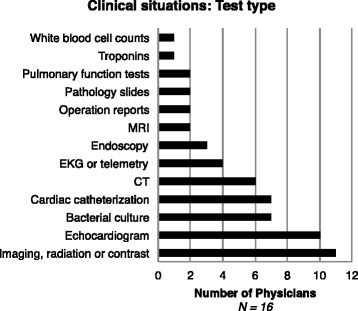
Response distribution to the question “In which specific (test type) clinical situations would timely OI influence your medical decisions?” Abbreviations: MRI, magnetic resonance imaging; EKG, electrocardiogram; CT, computed tomography

Specific test-related clinical drivers for OI requests are presented in Fig. 4. Responses included imaging and laboratory tests. Imaging was the most frequently requested test, indicated by 11 of the 16 interviewed physicians. Specifically CT scan was identified by 6 physicians and magnetic resonance imaging (MRI) was identified by 6 physicians. Echocardiograms, cardiac catheterizations, electrocardiograms and troponin levels were mentioned by 10, 7, 4 and 1 of the 16 interviewed physicians, respectively. Bacterial cultures from urine, blood, or other sources were recognized as important to clinical decision-making by 7 physicians. Physicians also wanted specific information about blood cultures including speciation, antibiotic susceptibility and amount of bacteria present. Without this information, tests may need to be repeated and effective treatment is delayed or unnecessary treatment is provided.

**Fig. 5:**
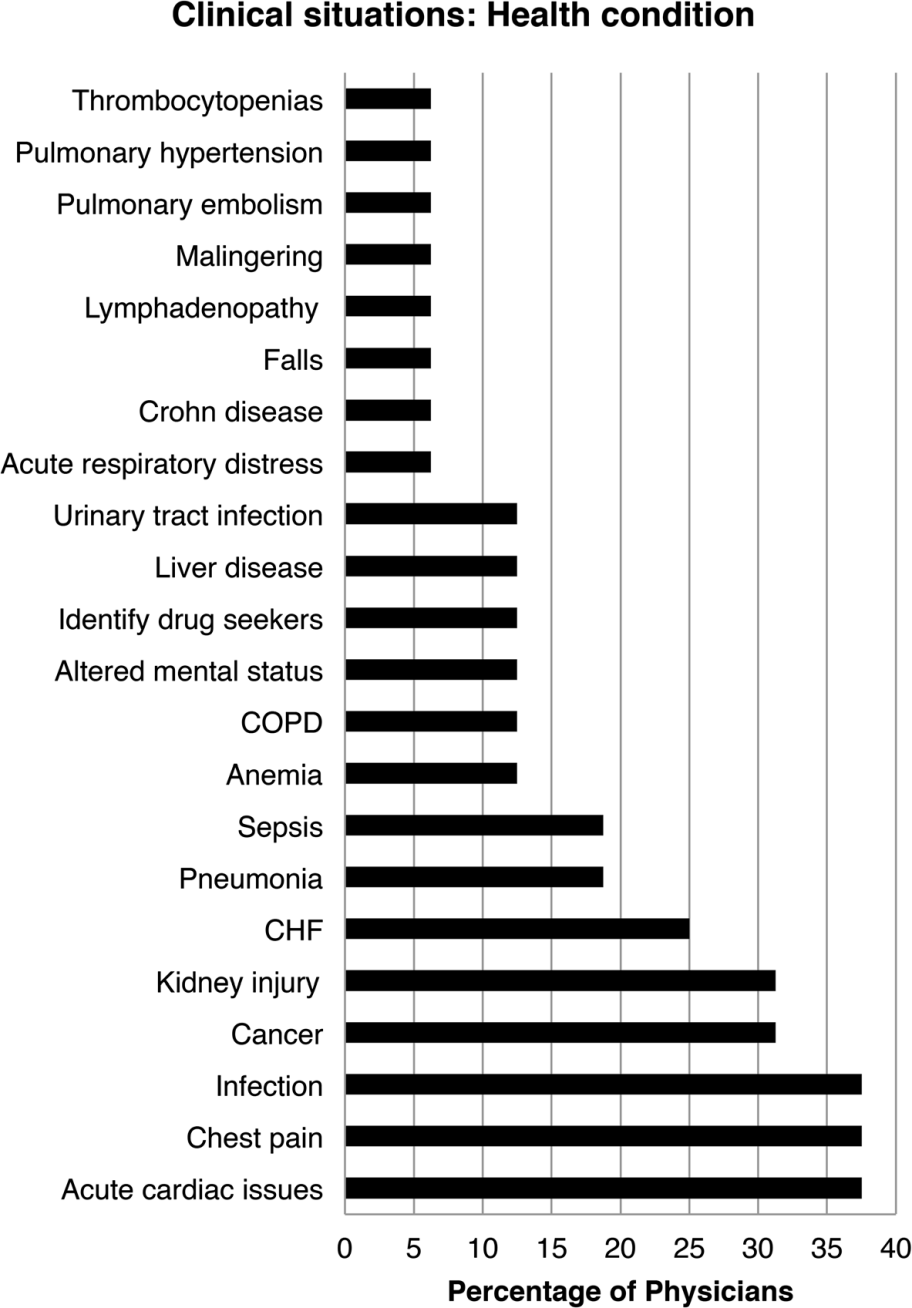
Response distribution to the question “In which specific (health condition) clinical situations would timely OI influence your medical decisions?” Abbreviations: ICU, intensive care unit; COPD, chronic obstructive pulmonary disease; CHF, congestive heart failure

Figure 5 shows the diverse health conditions that were identified as influential on medical decisions. The most frequently identified conditions were chest pain, acute cardiac conditions and infection, followed by kidney injury and cancer. 19 % of physicians discussed pneumonia and sepsis. Anemia was mentioned by 13 % of the interviewees. The remaining diagnoses were: thrombocytopenia, pulmonary hypertension, pulmonary embolism, malingering, lymphadenopathy, falls, Crohn’s disease, acute respiratory distress, urinary tract infection, liver disease, identifying drug-seekers, altered mental status and chronic obstructive pulmonary disease.

Other critical clinical drivers for OI were admissions to the intensive care unit (ICU) and transfers from other hospitals. 19 % of physicians identified critically ill patients as key examples of when OI would be valuable. The physicians elaborated that knowing the prior work-up of a critically ill patient can expedite life-saving patient care decisions. Studies have shown that patients unable or unwilling to communicate their health status, which is common in the ICU, are targets for using HIE [26]. Additionally, patients transferred from other hospitals are an important population because they are often sicker patients with complex medical conditions. Information about the workup done at the originating hospital is critical to the receiving providers to provide effective care to the patient. Unfortunately, transitions of care are difficult in these situations because of the emergent nature and abundance of information. In our interviews, 50 % of the physicians recognized “hospital transfers” as an opportunity for using HIE, which is consistent with other reports [36]. Six interviewees identified that they frequently get incomplete OI in these cases, and five interviewees said there was poor communication with transfers.

### Perceptions on pre-HIE electronic viewing of OI and potential for HIE

After discussion about situations where OI was influential in medical decisions, we wanted to explore how physicians physically interact with the outside records received. At the study hospital, outside documents are scanned into the EMR when they are received by fax, where they can then be viewed electronically. The original paper documents are stored in the patient’s bedside chart for temporary access. Physicians were asked, “Do you view the majority of the outside records in paper or electronic format? What percentage?”. Then, a discussion was generated about the positives and negatives of viewing each format. Physicians responded that they view OI electronically less than 40 % of the time. The negative aspects identified for electronic viewing were “excessive clicking” and “it does not facilitate parallel tasking”. Because there is limited screen space, it is difficult to view the outside documents while viewing current clinical information. Therefore, it is cumbersome to compare lab values or incorporate data into current documentation. Also, because of excessive amounts of records received and needing to adjust the zoom frequently to view content properly, the process requires extensive clicking. One of the benefits of electronic viewing was “remote access to records”.

At the end of the interviews, we explored physicians’ perceptions about HIE implementation in the future. Most physicians regarded HIE implementation positively; of the total number of responses to their perceptions about HIE, 85 % of the answers were coded as “positive”. Most providers recognize the need for universal access to patient records and anticipate streamlined patient care. The most frequent positive responses were that HIE will “facilitate better patient care”, lead to “less test redundancy” and “reduce costs”. Some other perceptions were that HIE will “reduce patient harm”, “decrease delays” and “improve transitions of care.” One physician mentioned that it would only be “beneficial if done the right way.” The negative feelings towards HIE were “concerns with HIPAA”, “access to meaningless data” and “slow down patient care”. This largely positive perception of the potential for HIE is an interesting contrast to providers that have experienced the problems of HIEs after implementation.

## Discussion

Our study suggests that the drivers for HIE utilization are the treatment of complex patients with a high number of comorbidities or with frequent previous healthcare visits, consistent with previous research [27]. Our study identifies the difficulties faced by physicians in an IM Department in a large hospital in order to obtain outside information prior to HIE implementation and provides a user needs assessment to inform HIE design and implementation. Our research begins to address the gap identified by O’Malley and colleagues between the policy makers’ expectations and the clinicians’ experiences with HIE [37]. We identified information that is important to physicians in specific clinical situations. Finally, we provided physicians’ insight into their perceptions of future implementation of HIE.

### User needs assessment to inform HIE design

Our results suggest that efficient organization of data shared by HIE is paramount to effective use. Prior data showing low usage by providers may be partly due to the user-unfriendly nature of current HIE, which were designed without empiric *a priori* end-user input. Table 4 presents a design for the implementation of HIE informed by the results of our study. By identifying patterns in responses by the physicians, we were able to start creating networks of clinical drivers and important information needs to inform medical decision-making. An example clinical domain is congestive heart failure. Many physicians identified congestive heart failure as a condition in which specific OI, such as echocardiograms, electrocardiograms and weight measurements, likely influence clinical decisions and patient outcomes. This finding from the interviews is particularly important because the Centers for Medicare and Medicaid Services (CMS) require all congestive heart failure patients to have an up-to-date echocardiogram documented [38]. One of our recommendations is having visual indicators that alert the user when OI in the HIE is relevant to specific diagnoses within the local system. For example, if a provider were treating a patient with heart failure, the HIE would indicate that an echocardiogram is available from an outside hospital. These clinically relevant features of an HIE would promote provider satisfaction by facilitating their HIE interface experience and potentially improve compliance with quality measures.

**Table 4:** Design recommendations for health information exchange in an Internal Medicine Department in a public hospital

Design recommendations
1. Allow keyword search functionality in OI
2. Provide the telephone number of the OI source for follow up questions
3. Provide the list of previous medications for medication reconciliation
4. Facilitate remote access to patients’ medical records
5. Provide computer screens that facilitate parallel tasking while reviewing documents electronically
6. Visual indicators for when OI is potentially relevant to specific diagnoses
7. Provide 1-click access to imaging, echocardiograms, bacterial cultures, cardiac catheterizations and CTs results (not only reports)
8. Prioritize OI access to patients with acute cardiac issues, chest pain, infection, cancer, and kidney injury
9. Prioritize OI access for hospital transfers and ICU patients

*OI* outside information, *CT* computerized tomography, *ICU* intensive care unit

### Problems amenable to HIE and factors that will remain problematic

Our analysis of physician interviews identified problems amenable to HIE and factors that will remain problematic despite HIE implementation. Some factors that will be alleviated by HIE are the physician not requesting OI because they assume the process will take too long or yield incorrect information. The current fax based system is inefficient, so often providers proceed with less information. However, a well designed HIE could provide some information faster and more reliably. This will be helpful especially in critical situations, such as the ICU or hospital transfers. Another factor amenable to HIE is when the patient does not know from where to request OI. In some HIEs, the provider will be able to see the location of all OI. Also, the difficult process to find more information after initial review of OI will be mitigated because the provider will not need to fill out request forms, fax them again, and wait for their return (See Figs. 1 and 2). They will only require re-accessing HIE to find more information. The problem of not being able to get OI after office hours will be eliminated as the HIE will be automated without relying on personnel to manually fax information.

Some problematic factors that will remain despite HIE implementation are if the OI is old information and needs to be repeated despite having easy access to it. HIE will also be challenged by an abundance of unorganized information received if it is not designed properly. Viewing original radiology imaging may be slow using HIE, so the need for imaging disks may not be alleviated by HIE completely. There may still be skepticism of the results from outside facilities, which will lead to repetitive testing. Similarly, the HIE will only have final reports for bacterial cultures and there may still be doubt as to the laboratory techniques for certain results (i.e., which location cultures were drawn from).

### Limitations & future work

Our study has limitations. First, the semi-structured interviews were a very powerful approach to obtain even subtle perceptions from the people who are involved in the process of requesting OI. However, by directly interviewing physicians, we are disturbing the environment and therefore the responses may be influenced by the presence of the research team. Second, because of the sample size and the specific setting (a teaching hospital using Epic), the conclusions obtained in this study may not be generalizable. However, this study represents an advance in the community of HIE knowledge since this research has not been carried out before in IM Departments within a hospital. Additionally, as of March 2015, Epic Systems is one of the top three EMR vendors comprising nearly 60 % of the market share of primary certified EMRs [39]. Future research should be done using a longitudinal approach, and ideally a larger number of settings. Finally, we also had attrition bias due to non-responses and we did not address any potential confounding due to user characteristics. For example, the level of computer skills may have biased physicians’ responses. Nonetheless, all the interviewees had at least 2.5 years of experience in the same IM Department and with Epic.

There are various aspects that can be addressed in future work. First, the effect of provider access to clinically relevant OI on length of stay and resource utilization should be assessed. Linking OI to patient outcomes is key to demonstrating HIE value. Second, patients with abdominal pain and cardiac problems should be specifically explored since these patients represent a large amount of OI requests. Third, HIE research should focus on ICU patients or hospital transfer admissions, as others have explored the challenges of communication between hospitalists and primary care physicians [40].

## Conclusion

By using mixed-methods we were able to map the current process of requesting OI, define provider perceptions, and compare those perceptions to quantitative data. This knowledge provides a user needs assessment for informing future HIE design and implementation. Further, our study combined with other research can direct future financial incentives to specifically promote evidence-based functionality that improves important outcomes. As meaningful use has improved EMR adoption, incentives for HIE paired with physician-guided implementation can likely improve the utilization of HIE.

## Additional file

Additional file 1:
**Semi-structured interview: list of close- and open-ended questions used during the semi-structured interviews.** (DOCX 23 kb)

## Abbreviations

CHF: Congestive heart failure
CMS: Centers for Medicare and Medicaid Services
COPD: Chronic obstructive pulmonary disease
CT: Computerized tomography
EKG: Electrocardiogram
EMR: Electronic medical record
HIE: Health information exchange
ICU: Intensive care unit
IM: Internal medicine
MRI: Magnetic resonance imaging
OI: Outside clinical information
