# Supplementary material for: A user needs assessment to inform health information exchange design and implementation
Source: BMC Med Inform Decis Mak. 2015 Oct 12;15:81. doi: 10.1186/s12911-015-0207-x (PMC4603345; doi:10.1186/s12911-015-0207-x)
Supplement: Additional file 1: — Semi-structured interview: list of close- and open-ended questions used during the semi-structured interviews. (DOCX 23 kb) [file 12911_2015_207_MOESM1_ESM.docx]

**Additional Files:** Semi-structured interview

Our semi-structured interview was design to have duration of 30 minutes. The questions included in the interview are as follow.

# Demographic information

## What is your job category? (e.g., attending or resident.)

# On the fax-based process of requesting outside records

## Validate the current process of requesting outside record, and identify the problems that you usually experience the process

## How many patients do you request for outside records on average (per week)? What percentage?

## In which situations do you know outside information exists but you do not request for records? What percentage?

## Do you view the majority of the outside records in paper or electronic format? What percentage?

## How many hours or days do you wait for outside records (from request to viewed)?

## Give examples in which outside information were requested and you encountered problems. What percentage?

# On the HIE-based process of requesting outside records

## In which specific clinical situations would timely outside information influence your medical decisions?
